# Editorial: Advanced Carbon Chemistry for Rechargeable Batteries

**DOI:** 10.3389/fchem.2020.00667

**Published:** 2020-09-08

**Authors:** Hongshuai Hou, Weijie Li, Weihua Chen, Wei Tang

**Affiliations:** ^1^Central South University, Changsha, China; ^2^University of Wollongong, Wollongong, NSW, Australia; ^3^Zhengzhou University, Zhengzhou, China; ^4^Xi'an Jiaotong University, Xi'an, China

**Keywords:** carbon materials, batteries, electrochemistry, energy storage, energy chemistry

The growing demand for green, alternative, and sustainable energy technology greatly expedites the development of energy storage/conversion devices like rechargeable batteries and supercapacitors. Carbon-based materials, which serve as a prevalent candidate in rechargeable batteries, have been widely explored due to their abundance, non-toxicity, stability, and durability. Furthermore, in other types of anode materials (including alloys, metal oxides, and metal sulfide), the introduction of carbon heterophase nanocomposite has been considered a prevalent strategy in enhancing the cycling performance due to an integrated structure stability during the charging/discharging process.

Amorphous carbon literally represents carbon with a low graphitization degree. The long-term disordered and short-term ordered structure is a typical feature for amorphous carbon in terms of micro texture. Specifically, amorphous carbon comprised of *sp*2 hybridized carbon which is rich in suspending bonds and other structural defects. Compared with graphite materials, amorphous carbon anodes exhibit a larger specific surface area, an abundance of porous structures and more active sites, which could bring about improved electrochemical properties.

When applied in rechargeable batteries, the microstructure and morphology, the graphitization degree, the specific surface area, the surface functionality, and porosity of carbon materials work synergistically to affect electrochemical performances. The unmodified bulk carbon used in anode material suffers from poor electrochemical performances due to the limited electron transport, sluggish ion migration, and undeveloped active sites. Consequently, in order to enhance the reversible capacity and electrochemical performance of carbon-based anode materials, the rational design and modification for advanced carbon-based materials has been proposed.

Heteroatom doping (B, N, P, S) has been regarded as a promising method to enhance the electrochemical properties of the carbon electrode. Heteroatom doping of large radius atoms (P, S) increases the interlayer spacing and induces structural distortion, providing more active sites for Li^+^, Na^+^, and K^+^ storage. Heteroatom doping for small diameter atoms (B, N) may adjust the electron cloud density to facilitate the charge transfer and enhance the electrode-electrolyte interactions, and prove the wettability of the surface. In this topic collection, P/N-co-doped carbon nanosheets, N-doped graphene, and S-doped hard carbon@rGO composites for rechargeable batteries are reported, the chemical mechanism and process are discussed systematically. In addition, defects, pore structure, and micromorphology of carbon materials on the electrochemical performances are discussed as well.

Carbon materials are often utilized as “additives” to improve the electrochemical performances of other electrode materials, such as metal oxides/sulfides/phosphides. The introduction of carbon materials can not only enhance the conductivity but also accommodate the volume change of electrode materials, which leads to the improved cycle stability and rate capability. In this topic collection, silicon, FeOOH, CuCo_2_S_4_, and Cu_3_P modified by functional carbon materials are reported.

We hope it will be helpful for readers to further understand the advanced carbon chemistry for rechargeable batteries.

## Author Contributions

HH, WL, WC, and WT co-edit this Research Topic. All authors contributed to the article and approved the submitted version.

## Conflict of Interest

The authors declare that the research was conducted in the absence of any commercial or financial relationships that could be construed as a potential conflict of interest.

